# Impact of statin treatment on cardiovascular risk in patients with type 1 diabetes: a population-based cohort study

**DOI:** 10.1186/s12967-023-04691-6

**Published:** 2023-11-12

**Authors:** Joonsang Yoo, Jimin Jeon, Minyoul Baek, Sun Ok Song, Jinkwon Kim

**Affiliations:** 1 Department of Neurology, Yongin Severance Hospital, Yonsei University College of Medicine, 363 Dongbaekjukjeon-daero, Giheung-gu, Yongin-si, Gyeonggi-do 16995 Republic of Korea; 2 Division of Endocrinology and Metabolism, Department of Internal Medicine, National Health Insurance Service Ilsan Hospital, 100 Ilsan-ro, Ilsandong-gu, Goyang, 10444 Republic of Korea

**Keywords:** Type 1 diabetes mellitus, Cardiovascular disease, Cohort study, Statin

## Abstract

**Background:**

Cardiovascular disease (CVD) is a major complication in type 1 diabetes mellitus (T1D) patients. Previous studies have suggested that statins may be helpful for prevention of CVD in T1D, but there are limited data on the role of statins in T1D. We investigated the relationship between statin treatment and cardiovascular risk in T1D patients using a population-based cohort.

**Methods:**

We conducted a retrospective cohort study using the Korean nationwide health insurance database from January 2007 to December 2017. This study included 11,009 T1D patients aged ≥ 20 years without a prior history of CVD. The primary outcome was a composite development of stroke or myocardial infarction. Statin use during follow-up was treated as a time-varying variable. We performed a multivariable time-dependent Cox regression analysis adjusting for sex, age, type of insurance, hypertension, renal disease, and use of antiplatelets and renin–angiotensin–aldosterone system inhibitors.

**Results:**

During the mean follow-up of 9.9 ± 3.7 years of follow-up, 931 T1D patients (8.5%) suffered primary outcome. Statin treatment was associated with a reduced risk of the primary outcome (adjusted hazard ratio, 0.76; 95% confidence interval 0.66–0.88; p < 0.001). Statin use led to decreased risks of ischemic stroke and myocardial infarction, but was not related to hemorrhagic stroke. We also found that the risk of cardiovascular events decreased as the cumulative exposure duration of statins increased.

**Conclusions:**

Statin use was associated with a lower risk of cardiovascular events in T1D patients. Further prospective studies are needed to confirm the potential role of statins in prevention of CVD in patients with T1D.

## Introduction

Type 1 diabetes mellitus (T1D) is a chronic metabolic disease precipitated by an immune-associated destruction of insulin-producing β-pancreatic cells [1]. Abnormally high blood glucose level in T1D affects major organs and can lead to a variety of complications over time. Typically, people with T1D have a greater risk of cardiovascular events than the general population, and cardiovascular disease (CVD) is a major cause of morbidity and mortality in those with T1D [2]. Therefore, lifelong control of the cardiovascular risk profile is essential in the management of individuals with T1D.

Statins are a class of lipid-lowering medications and one of the most widely used drugs worldwide. With good efficacy and well-established safety, statins remain the cornerstone in the prevention and treatment of CVD [3, 4]. In type 2 diabetes mellitus (T2D), statin treatments have beneficial effects on the prevention of CVD and mortality [5]. Contrary to the cumulative evidence supporting the use of statins in people with T2D for cardiovascular prevention, there are very limited data concerning the use of statins in T1D [6]. Regarding the high CVD risk in T1D, guidelines recommend statin treatment for primary prevention in T1D patients > 40 years of age [7, 8]. However, these recommendations are mainly derived from studies on patients with T2D, and the effect of statins on cardiovascular risk in T1D patients is not well established. Especially, knowledge of the role of statins in Asian patients with T1D is lacking [9]. In the current study, we investigated the association between statin use and the development of CVD in T1D using a population-based cohort from the Korean nationwide healthcare claims database.

## Methods

### Data source

This study is a retrospective analysis of a population-based T1D cohort from a nationwide health insurance claims database in Korea. Korea has a public single-payer health insurance system that covers the entire nation, and the Health Insurance Review and Assessment Service (HIRA) is a government agency specializing in reviewing medical claims from health care providers and quality assessment of health care services [10]. For the purpose of political and academic research with an appropriate review process, HIRA provides health care claims data to researchers. The HIRA database contains health care information from each patient visit to a medical institution (primary-care clinics, public health centers, general hospitals, and tertiary referral hospitals), diagnoses, prescriptions, medical procedures, and demographic data. In the claims data, the diagnoses at each hospital visit are recorded according to the 10th edition of the International Statistical Classification of Diseases (ICD-10) coding scheme, and prescription records include drug name, dose, prescription date, and duration. The provided claims dataset is anonymized and does not contain any identifiable information. This study was approved by the Institutional Review Board of Yongin Severance Hospital, Yonsei University College of Medicine (9-2021-0119). The requirement for informed consent in this study was waived because of its retrospective nature, and analyses were performed using fully anonymized data.

### Study cohort with T1D

**Fig. 1:**
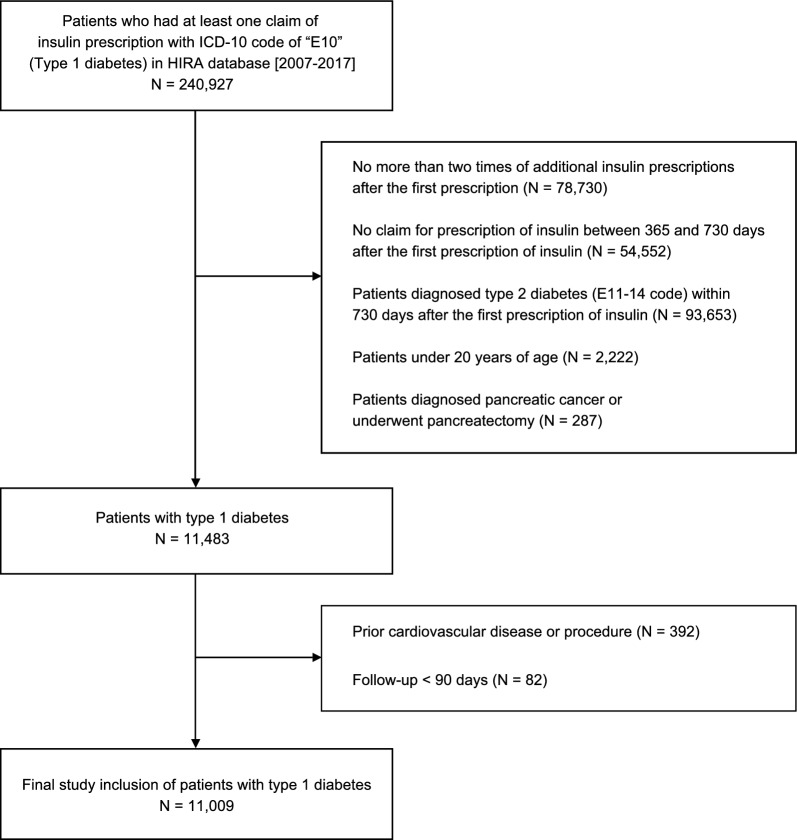
Flow chart of patient enrollment

Using population-based healthcare claims data from the HIRA, we selected patients who received an insulin prescription with a diagnostic code of T1D (ICD-10 code of “E10”) between 2007 and 2017. Because T1D is often confused with other types of diabetes mellitus, such as T2D, we tried to identify T1D patients using strict criteria from a previous study of T1D in Korea [11]. According to the criteria, we only included patients with ≥ 3 claims for prescription of insulin. Patients without an additional insulin regimen established between 1–2 years after the first insulin treatment were excluded. Patients who had a diagnosis of another type of diabetes (“E11–14”) within 2 years of the first insulin prescription were also excluded. We also excluded patients < 20 years of age in whom the effect of statins is uncertain. Patients with pancreatic cancer or who underwent total or partial pancreatectomy were excluded. Additionally, those who had CVD prior to T1D diagnosis (ischemic heart diseases: “I20–25”, stroke: “I60–64, I69”, carotid artery stent, carotid endarterectomy, coronary stent insertion, coronary artery bypass graft) and those with a follow-up period < 90 days were excluded. Figure 1 demonstrates the inclusion and exclusion of study participants.

### Follow-up and study outcomes

The index date and start date of follow-up were defined as the initial date of insulin prescription with the diagnostic code of “E10” for T1D in each patient. Patients with T1D included in the study were followed up to the occurrence of the primary outcome; censoring; or until December 31, 2020 (the study end date). The primary outcome was development of a cardiovascular event and was a composite of stroke and myocardial infarction (MI)—whichever occurred first. Stroke was determined by admission with the primary diagnosis of “I60–63” with brain computed tomography or magnetic resonance imaging performed during the admission [12]. MI was defined by admission with a primary diagnosis of “I21”. Diagnostic accuracies for stroke and MI based on the health claims data in Korea have been reported as sufficient [13, 14]. The secondary outcome was ischemic stroke (“I63”), hemorrhagic stroke (“I60–62”), or MI (“I21”), which are components of the primary outcome. In the analysis for secondary outcomes, individual outcomes were treated as competing events, and patients experiencing competing events were censored at the time the event occurred.

### Covariates

We collected data on demographics, such as age, sex, type of insurance (national health insurance and medical aid from the government), and presence of hypertension and renal disease from the HIRA database. The public health care system in Korea is a two-tiered system of national health insurance and medical aid. The Korean medical aid program provides free or reduced-cost care for low-income families and individuals. The remaining proportion of the population is covered by national health insurance. The presence of hypertension was considered if the patient received anti-hypertensive agents and had the corresponding diagnostic codes of hypertension (ICD-10 codes “I10–13”, “I15”) [15]. Renal disease was identified by the presence of relevant diagnostic codes (ICD-10 codes “N17–19”, “E10.2” or “I12–13”) or claims of hemodialysis, peritoneal dialysis, and/or procedures related to renal disease [12, 16].

### Use of statins, antiplatelets, and RAAS inhibitors

**Fig. 2:**
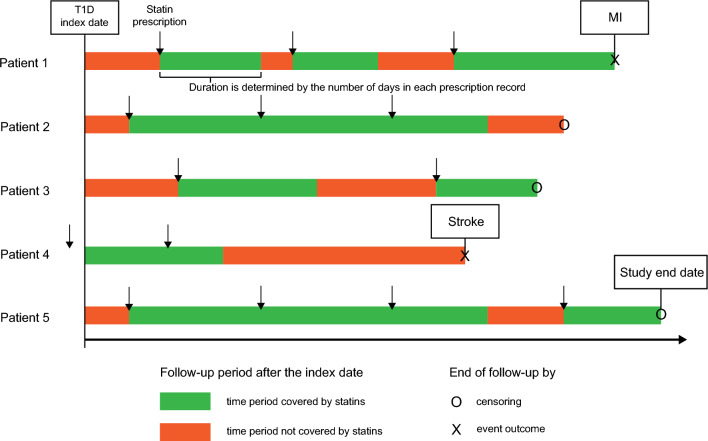
An example of determining the prescription of a statin as a time-dependent variable

During the study period, we collected prescription data (drug name, dose, and duration) from the HIRA database for statins (atorvastatin, fluvastatin, lovastatin, pitavastatin, pravastatin, rosuvastatin, and simvastatin) and oral antiplatelets (aspirin, clopidogrel, ticlopidine, ticagrelor, prasugrel, triflusal, dipyridamole, and cilostazol) in each patient. Because medication intake varies over time, treatments involving these medications during the follow-up period have a time-varying feature. Additionally, we assessed the use of renin–angiotensin–aldosterone system (RAAS) inhibitors, such as angiotensin-converting enzyme inhibitors and angiotensin receptor blockers, in each patient throughout the follow-up period. On each day of the follow-up period, the use of medications was determined by prescription coverage (Fig. 2). In the analyses, the use of medications (statins, antiplatelets, and RAAS inhibitors) was treated as a time-dependent variable.

### Statistical analyses

Characteristics are expressed as mean ± standard deviation values for continuous variables and number (%) for categorical variables. To evaluate whether treatment with statins was associated with the occurrence of a subsequent stroke or MI, we calculated hazard ratio (HR) and 95% confidence interval (CI) values based on a time-dependent Cox proportional hazards regression model for the development of cardiovascular events, which included the use of statins as a time-dependent variable. Adjustments were performed for sex, age, type of insurance, presence of hypertension and renal disease, use of antiplatelets and RAAS inhibitors. The assumption of proportional hazards for use of statins in the Cox regression model was tested by calculating the Schoenfeld residuals using the “*cox.zph*” function in the R package of “*survival*” and was satisfactory. To evaluate the potential interactions with statin treatment, we performed subgroup analyses according to sex, age group (20–39 or ≥ 40 years), and enrollment period.

As an additional sensitivity analysis, we conducted a nested case–control study with the T1D cohort [17]. In this design, cases are patients who experienced the primary outcome during the follow-up period. For each case, we matched three controls with replacements from the cohort (1:3 matching) who were free from the event at the time of the primary outcome in their matched case by incidence density sampling. Cases and controls were matched for all collected variables except the use of statins (sex, age [± 1 year], type of insurance, presence of hypertension, renal disease, use of antiplatelets and RAAS inhibitors). Conditional logistic regression analysis was performed with the matched case–control groups to estimate the odds ratio (OR) and 95% CI for the primary outcome according to statin treatment. We also evaluated the risk for the primary outcome according to cumulative exposure duration to statins instead of the use of statins. The cumulative exposure duration to statins was calculated as the sum of days of statin treatment between the index date and the time of the primary outcome, which was subdivided into three categories of < 1 year, 1–3 years, and > 3 years. We used the group of cumulative exposure duration to statins < 1 year as the reference category to investigate the results for each duration group. Statistical analyses were performed using SAS (version 9.4; SAS Institute, Cary, NC, USA) and R software (version 3.3.3; The R Foundation for Statistical Computing, Vienna, Austria; http://www.R-project.org/). *P* values were two-sided, and *P* < 0.05 was considered statistically significant.

## Results

### Characteristics of the included T1D patients

According to the inclusion and exclusion criteria, 11,009 patients with T1D aged ≥ 20 years were included in this study (Fig. 1). Among the included T1D patients, 6785 (61.6%) were men, and the mean age at enrollment was 50.7 ± 15.2 years. For the type of health insurance, 805 patients (7.3%) were eligible for medical aid from the government, while 10,204 patients (92.7%) were covered by the National Health Insurance Service. Hypertension and renal disease were present in 4548 (41.3%) and 1508 (13.7%) patients, respectively (Table 1). At the index date, 7% of patients were taking statins, while 17% were doing so at 90 days. However, although this number increased over time, it did not reach 50% even 10 years after T1D diagnosis (Table 2).

**Table 1:** Baseline characteristics of included patients

Variable	Total (n = 11,009)
Sex, male	6785 (61.6)
Insurance type	
Health insurance	10,204 (92.7)
Medical aid	805 (7.3)
Age, years	50.7 ± 15.2
Comorbidities	
Hypertension	4548 (41.3)
Renal disease	1508 (13.7)
Index year	
2007–2008	6456 (58.6)
2009–2012	2950 (26.8)
2013–2017	1603 (14.6)

Data are represented as number of participants (%) or mean ± standard deviation

**Table 2:** Proportion of type 1 diabetes patients taking statins over time after cohort enrollment

	Index date	+ 90 days	+ 2 years	+ 4 years	+ 6 years	+ 8 years	+ 10 years
Statin users	773 (7.02)	1894 (17.20)	2220 (20.49)	2740 (27.20)	2968 (34.29)	2952 (38.87)	2561 (43.66)
Number at risk	11,009	11,009	10,832	10,072	8955	7595	5864

Data are number (%)

### Associations between statin treatment and cardiovascular events

During the mean follow-up period of 9.9 ± 3.7 years, 931 patients (8.5%) experienced the primary outcome of cardiovascular event. As a first cardiovascular event, 669 (71.9%) patients suffered stroke (ischemic stroke: 553 patients; hemorrhagic stroke: 116 patients), and 262 (28.1%) suffered MI. Among patients who experienced the primary outcome, 32.3% were taking statins at the time of the event. In multivariable time-dependent Cox analysis, statin treatment was significantly associated with decreased risk of cardiovascular events (adjusted HR, 0.76; 95% CI 0.66–0.88) (Table 3). During the secondary outcome analyses to identify the effects of statin on individual outcomes, statin use was significantly associated with low risk of ischemic stroke (adjusted HR, 0.74; 95% CI 0.61–0.89) and MI (adjusted HR, 0.74; 95% CI 0.56–0.96), whereas there was no association between statin use and hemorrhagic stroke (Table 4).

**Table 3:** Factors associated with occurrence of the primary outcome

Variable	Univariable HR [95% CI]	*P* value	Adjusted HR [95% CI]^a^	*P* value
Sex, male	1.16 [1.01–1.32]	0.035	1.38 [1.20–1.58]	< 0.001
Age, years	1.05 [1.05–1.06]	< 0.001	1.05 [1.04–1.05]	< 0.001
Insurance type				
Health insurance	1 (Ref)	–	1 (Ref)	–
Medical aid	1.35 [1.08–1.69]	0.009	1.35 [1.07–1.70]	0.010
Comorbidities				
Hypertension	2.04 [1.79–2.32]	< 0.001	1.26 [1.08–1.48]	0.004
Renal disease	1.32 [1.10–1.59]	0.003	1.18 [0.98–1.43]	0.080
Medication				
Antiplatelet	1.81 [1.59–2.07]	< 0.001	1.32 [1.14–1.53]	< 0.001
RAAS inhibitor	1.36 [1.19–1.56]	< 0.001	0.89 [0.76–1.04]	0.131
Statin	0.96 [0.83–1.10]	0.531	0.76 [0.66–0.88]	< 0.001

Data were obtained from a multivariable time-dependent Cox proportional hazard regression model

*CI* confidence interval, *HR* hazard ratio, *RAAS* renin–angiotensin–aldosterone system

^a^Adjusted for sex, age, insurance type, hypertension, renal disease, and medications

**Table 4:** Secondary outcome analysis according to statin treatment

Medication	Adjusted HR [95% CI]^a^			
	All stroke	Ischemic stroke	Hemorrhagic stroke	Myocardial infarction
Number of events	669	553	116	262
Statin	0.77 [0.65–0.92], *P* = 0.003	0.74 [0.61–0.89], *P* = 0.002	0.96 [0.65–1.42], *P* = 0.854	0.74 [0.56–0.96], *P* = 0.025

Data were obtained from a multivariable time-dependent Cox proportional hazard regression model

*HR* hazard ratio, *CI* confidence interval

^a^Adjusted for sex, age, insurance type, hypertension, renal disease, and treatment with antiplatelets and renin–angiotensin–aldosterone system inhibitors

**Fig. 3:**
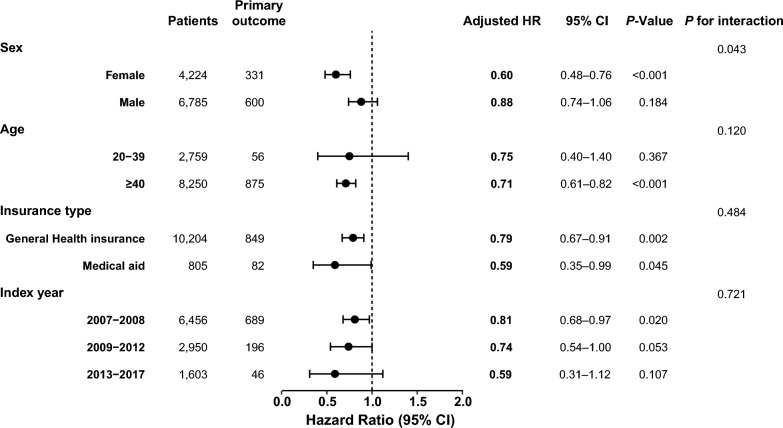
Subgroup analysis of cardiovascular disease occurrence according to statin treatment

In subgroup analyses (Fig. 3), the beneficial effect of statins was present in both male (adjusted HR, 0.88; 95% CI 0.74–1.06) and female (adjusted HR, 0.60; 95% CI 0.48–0.76), but the statistical significance was only found in female. Both the 20–39-year-old (adjusted HR, 0.75; 95% CI 0.40–1.40) and the ≥ 40-year-old (adjusted HR, 0.71; 95% CI 0.61–0.82) groups showed a tendency to be associated with a low rate of the primary outcome on statins, but statistical significance was present only in those ≥ 40-year-old. There was no significant interaction between the association of statin and low cardiovascular events according to the type of health insurance or enrolled year.

### Nested case–control study

In the nested case–control analysis performed as a sensitivity analysis, 675 cases (patients with the primary outcome) were matched to 2025 controls without the primary outcome using 1:3 incidence density sampling (Table 5). The cases and controls were fully matched according to baseline characteristics, the use of antiplatelets and RAAS inhibitors. The proportion of those taking statins was lower in the case group than in the control group (32.4% in the case group vs. 38.4% in the control group). In the conditional logistic regression analysis, statin treatment was significantly associated with a lower risk of cardiovascular events (OR, 0.73; 95% CI 0.59–0.89). When we evaluated the risk according to the duration of cumulative exposure to statins, as the amount of cumulative exposure to statins increased, the risk of cardiovascular events decreased (1–3 years: OR, 0.73 [95% CI 0.57–0.94] and > 3 years: OR, 0.60 [95% CI 0.47–0.77] compared to < 1 year). The dose–response association between longer cumulative exposure to statins and lower risk was seen for ischemic stroke and myocardial infarction, but not for hemorrhagic stroke (Table 6).

**Table 5:** Characteristics of cases and matched controls in the nested case–control study

Variable	Case n = 675	Control n = 2025	Odds ratio [95% CI]	*P* value
Sex, male	400 (68.1)	1,380 (68.1)	Matched	
Age, years	57.8 ± 10.4	57.8 ± 10.4	Matched	
Insurance type			Matched	
Health insurance	668 (99.0)	2004 (99.0)		
Medical aid	7 (1.0)	21 (1.0)		
Comorbidities				
Hypertension	352 (52.1)	1056 (52.1)	Matched	
Renal disease	39 (5.8)	117 (5.8)	Matched	
Medication				
Antiplatelet	261 (38.7)	789 (38.7)	Matched	
RAAS inhibitor	302 (44.7)	906 (44.7)	Matched	
Statin	219 (32.4)	778 (38.4)	0.73 [0.59–0.89]	0.002
Cumulative statin exposure				
< 1 year	431 (63.9)	1129 (55.8)	1 (Ref)	
1–3 year(s)	103 (15.3)	352 (17.4)	0.73 [0.57–0.94]	0.015
> 3 years	141 (20.9)	544 (26.9)	0.60 [0.47–0.77]	< 0.001

*CI* confidence interval, *RAAS* renin–angiotensin–aldosterone system

**Table 6:** Risk of individual outcome according to treatment with statin in the nested case–control study

Variable	Primary outcome^a^	Secondary outcome			
		All stroke	Ischemic stroke	Hemorrhagic stroke	Myocardial infarction
Statin	0.73 [0.59–0.89], *P* = 0.002	0.81 [0.63–1.03], *P* = 0.083	0.76 [0.58–0.99], *P* = 0.039	1.12 [0.62–2.00], *P* = 0.710	0.56 [0.38–0.82], *P* = 0.003
Cumulative statin exposure					
< 1 year	1 (Ref)	1 (Ref)	1 (Ref)	1 (Ref)	1 (Ref)
1–3 year(s)	0.73 [0.57–0.94], *P* = 0.015	0.80 [0.60–1.07], *P* = 0.131	0.82 [0.60–1.12], *P* = 0.219	0.69 [0.33–1.48], *P* = 0.345	0.58 [0.35–0.95], *P* = 0.031
> 3 years	0.60 [0.47–0.77], *P* < 0.001	0.66 [0.49–0.88], *P* = 0.005	0.63 [0.45–0.87], *P* = 0.005	0.81 [0.41–1.58], *P* = 0.531	0.46 [0.29–0.74], *P* < 0.001

Data are expressed as odds ratio [95% confidence interval] obtained from conditional logistic regression analyses using the nested case–control dataset matched for sex, age, insurance type, comorbidities, and treatment with antiplatelets and renin–angiotensin–aldosterone system inhibitors

^a^Composite of stroke or myocardial infarction

## Discussion

In this population-based T1D cohort study, we evaluated the risk of CVD according to statin treatment. The number of 11,483 adult T1D patients is in line with the estimate of the registry study conducted in Korea [11]. In patients with T1D, treatment with statins was associated with a 24% lower risk of CVD. The association between statin use and fewer cardiovascular events was consistent in sensitivity analysis with a nested case–control design, and we also observed that fewer cardiovascular events occurred over a longer period of statin treatment.

It is well known that cardiovascular risk is increased in T1D patients [18]. Indeed, in our cohort study with 11,009 T1D patients, approximately 1 in 12 without previous CVD experienced a stroke or MI during the 10 year follow-up period. Several studies have demonstrated that development of cardiovascular complications is common in T1D patients, and the risk of CVD in T1D is greater than that in T2D patients [19, 20]. A cohort study conducted in the United Kingdom reported a 3.6- to 7.7-fold increase in major CVD in T1D patients compared to the general population [21]. Cardiovascular mortality in T1D patients is higher than that both in the general population and in T2D patients [19, 22]. Currently, CVD remains the leading cause of morbidity and mortality in T1D patients [23–25]. Considering the relatively early onset of T1D patients compared to T2D patients, development of CVD in T1D patients leads to more life-years lost [26].

The mechanism of high CVD risk in T1D is not fully understood, but long-term exposure to hyperglycemia, oxidative stress, and low-grade inflammation are characteristics of T1D and can contribute to the development and progression of vascular complications [27]. T1D is associated with a higher prevalence and more rapid progression of coronary atherosclerosis [28, 29]. Furthermore, the presence of both traditional and non-traditional cardiovascular risk factors is frequently confirmed in T1D patients, and metabolic syndrome is also commonly observed [30, 31]. Hyperglycemia due to a defect in insulin secretion in T1D also contributes to an increased risk of cardiovascular events [32].

Statins have been established to be beneficial for preventing cardiovascular events, which are major complications in T2D patients [33]. Based on the cumulative evidence, statin therapy is recommended for primary and secondary prevention of CVD in diabetic patients who are at greater risk [7]. Evidence from multiple large-scale randomized controlled trials of statin treatment suggests that the beneficial effect of statins on CVD is largely attributable to decrease of low-density lipoprotein cholesterol (LDL-C) [34]. In a study using Swedish national diabetes registry data, LDL-C was a significant predictor of death and CVD in patients with T1D [32]. For each 1 mmol/L increase of LDL-C level in T1D, there was a 35–50% greater CVD risk. Meanwhile, a low level of LDL-C in T1D was negatively associated with coronary atherosclerosis [35]. The relationship between the LDL-C–lowering effect of statins and a proportional reduction in CVD events is consistent between patients with T1D or T2D and non-diabetic individuals [36, 37]. In addition to lowering the LDL-C level, statins have multiple pleiotropic effects such as improving endothelial dysfunction, increasing nitric oxide bioavailability, inhibiting inflammatory responses, and stabilizing atherosclerotic plaques [38]. T1D patients have elevated levels of plasma markers, which reflect inflammation and endothelial dysfunction even before the clinical manifestation of macroangiopathy [39]. Elevated markers of inflammation and endothelial dysfunction are associated with a high risk of CVD in T1D patients [40, 41]. Administration of statins reduces the levels of inflammatory markers and improves endothelial dysfunction, although it is unclear whether statins have a similar effect in T1D [42].

There is a concern that the use of statins in T1D patients may adversely affect diabetes itself [43]. Concerns about impaired glycemic control and increased risk of diabetes with statin treatments are major discourages of adherent use of statins in clinical practice. In a study of T1D patients, statin use was associated with an increased level of HbA1c, reflecting the presence of impaired glycemic control [44]. A report also suggests that statins deteriorate insulin sensitivity in T1D patients [45]. Therefore, it is unclear whether regular statin use for the primary prevention of CVD is beneficial for T1D patients. In the current study, we demonstrated that statin use, particularly longer cumulative use, is associated with a lower risk of CVD. Our study suggests that the use of statins would assist with primary cardiovascular prevention in T1D patients at high risk. Whether the use of statins can promote hemorrhagic stroke is also a concern that inhibits statin use [46]. However, we did not find a significant relationship between the use of statins and hemorrhagic stroke in T1D patients.

In the subgroup analysis of the current study, the cardiovascular preventive effect of statins in T1D was more prominent in females than males. Currently, we did not have a clear answer whether this finding is coincidental or whether there is a notable sex difference in the effect of statins on T1D. One hypothesis is that the more prevalent risk factors, unhealthy lifestyles, and poor drug adherence in males might interrupt the beneficial effect of statins. Further study is needed for this topic. Statin treatment led to fewer cardiovascular events in both those ≥ 40 years and 20–39 years of age. However, this relationship was only significant in the ≥ 40 years age group. We suppose that this trend is due to a lack of statistical power in the younger age group, as most cardiovascular events occurred in participants ≥ 40 years of age. The current guideline for statin use in T1D patients is in accordance with the guidelines established for T2D patients, and it is recommended to use statins in T1D patients > 40 years of age and selectively use statins in those 20–39 years old according to cardiovascular risk [47]. However, the evaluation of individual cardiovascular risk is challenging [48, 49]. The role of statins for primary prevention in T1D patients aged 20–39 years is unclear; further studies are needed to establish whether statin therapy is beneficial in this patient group. In the current study, although the use of statins in T1D patients has increased over time, only one-third of patients were receiving statins at the time of the cardiovascular event. In patients with T1D, the use of statins was substantially less common than the guidelines suggested, and the difference is greater in view of primary prevention compared to secondary prevention for CVD [50, 51]. Given this low statin usage rate, clinicians need to more actively consider the use of statins for T1D patients and increase patient adherence to statins.

Our study has several limitations. First, because this was a retrospective study, there may be bias. Also, this study used a cohort derived from a single ethnic group. Since the characteristics of CVD and T1D may vary by country or ethnicity, caution is needed in generalizing the results. The use of health care claims data also produces limitations. We could not get clinical data such as the degree of control of diabetes (including HbA1c and glucose level) or the lipid profile of individual patients. We also did not know the indications for statin use; there is a possibility of statins being used only in patients with poor lipid profiles, but this could not be verified. Although strict criteria were used to accurately identify T1D patients, our dataset may include misdiagnosis or inadequate information due to the inherent limitation of health claims data. Based on the claims data and utilizing several criteria, patients with T1D were identified and an index date was established. However, this index date may differ from the onset of T1D. Finally, there might be a difference between the prescription records issued by physicians and the patients’ actual medication intake. However, several strengths highlight the significance of this study. Unlike many Western countries, Korea has a very low prevalence of T1D patients [11]. Therefore, we had to conduct this study using nationwide healthcare claims data. Using a population-based cohort, we were able to include a relatively large number of patients with T1D and evaluate long-term data to reveal the relationship between the development of CVD and statin treatment in T1D in real-life practice. In addition, to increase the strength of the research results, we reconfirmed the association between statin use and CVD in T1D patients by performing additional sensitivity analysis using a nested case–control study. We also identified a trend toward reduced CVD risk in T1D patients with a longer duration of statin treatment. In addition, we performed a subgroup analysis according to insurance status, which can indirectly reflect economic status, and confirmed that statin use is related to CVD risk regardless of insurance status. Our research data from an Asian T1D population consistently showed that statin treatment could contribute to CVD risk reduction in the high-risk group. In addition to the existing evidence that statin administration in T1D patients can contribute to CVD risk reduction [9], the present study provides supporting evidence for the current guideline recommending statin administration in T1D patients.

## Conclusions

In this nationwide T1D cohort study, the use of statins was associated with an ~ 25% reduction in CVD. Also, statins were being used less frequently than recommended in the guidelines. As the actual use of statins was not sufficient, more aggressive use of statins for CVD prevention in T1D patients should be considered. Further prospective studies are needed to confirm the results of this study.

## Data Availability

The dataset supporting the results of this study is accessible from HIRA in Korea, but with restrictions to data availability. The use of the dataset is restricted to the current research under license; therefore, public access of the dataset is not available. Researchers are only access the data upon reasonable request with approval from the inquiry committee of research support in HIRA (https://opendata.hira.or.kr/or/orb/useGdInfo.do).

## Abbreviations

T1D: Type 1 diabetes mellitus
CVD: Cardiovascular disease
T2D: Type 2 diabetes mellitus
HIRA: Health insurance review and assessment service
ICD-10: International statistical classification of diseases
MI: Myocardial infarction
HR: Hazard ratio
CI: Confidence interval
OR: Odds ratio
LDL-C: Low-density lipoprotein cholesterol
